# High Abundance of Thaumarchaeota Found in Deep Metamorphic Subsurface in Eastern China

**DOI:** 10.3390/microorganisms10030542

**Published:** 2022-03-01

**Authors:** Wenhui Zhang, Weiguo Hou, Xiangzhi Zeng, Shang Wang, Hailiang Dong

**Affiliations:** 1 Center for Geomicrobiology and Biogeochemistry Research, State Key Laboratory of Biogeology and Environmental Geology, China University of Geosciences, Beijing 100083, China; 2157190005@cugb.edu.cn (W.Z.); dongh@cugb.edu.cn (H.D.); 2 Institute of Geology, Chinese Academy of Geological Sciences, Beijing 100037, China; zeng.xz@163.com; 3 CAS Key Laboratory of Environmental Biotechnology, Research Center for Eco-Environmental Sciences, Chinese Academy of Sciences, Beijing 100085, China

**Keywords:** Thaumarchaeota, AOA, deep subsurface, biofilm, CCSD

## Abstract

Members of the Thaumarchaeota phylum play a key role in nitrogen cycling and are prevalent in a variety of environments including soil, sediment, and seawater. However, few studies have shown the presence of Thaumarchaeota in the terrestrial deep subsurface. Using high-throughput 16S rRNA gene sequencing, this study presents evidence for the high relative abundance of Thaumarchaeota in a biofilm sample collected from the well of Chinese Continental Scientific Drilling at a depth of 2000 m. Phylogenetic analysis showed a close relationship of these thaumarchaeotal sequences with known ammonia-oxidizing archaea (AOA) isolates, suggesting the presence of AOA in the deep metamorphic environment of eastern China which is believed to be oxic. Based on fluid geochemistry and FAProTax functional prediction, a pathway of nitrogen cycling is proposed. Firstly, heterotrophic nitrogen fixation is executed by diazotrophic bacteria coupled with methane oxidation. Then, ammonia is oxidized to nitrite by AOA, and nitrite is further oxidized to nitrate by bacteria within the phylum Nitrospirae. Denitrification and anaerobic ammonia oxidation occur slowly, leading to nitrate accumulation in the subsurface. With respect to biogeochemistry, the reaction between downward diffusing O_2_ and upward diffusing CH_4_ potentially fuels the ecosystem with a high relative abundance of Thaumarchaeota.

## 1. Introduction

Thaumarchaeota—the third designated archaeal phylum, previously considered to be a lineage of the phylum Crenarchaeota [1]—contains ammonia-oxidizing archaea (AOA) as well as deeply rooted non-AOA [2,3]. AOA catalyze the first and the rate-limiting step of nitrification—the aerobic oxidation of ammonia to nitrate via nitrite [2]—and are the most abundant archaea in terrestrial and marine systems [4]. Thus, AOA are thought to be key members of the global nitrogen and carbon biogeochemical cycles [4,5,6].

Oxygen availability and pH are hypothesized to be the key environmental parameters that drove diversification of the AOA over evolutionary time [3,7]. Likewise, the abovementioned environmental parameters also constrain the distribution of AOA. As such, the majority of studies on the AOA community have focused on topsoil where O_2_ is readily available to these microorganisms [8,9]. Constrained by O_2_ availability, AOA communities only reach a depth of a little more than 2 m in floodplain sediments [2], while in the semi-arid floodplains of the western United States, AOA have been recovered from a depth of more than 10 m [10]. These studies observed great variations in the AOA community over depth [2,10]. Beyond the parameter of O_2_, cultivation-based and cultivation-independent studies have extended the habitat of AOA from mesophilic environments to thermophilic environments, i.e., terrestrial hot springs [11,12,13,14,15,16,17].

The deep subsurface is hot, oligotrophic, and primarily rocky, thus it was thought to be hostile to microbial life. In actuality, the deep subsurface harbors substantial microbial biomass [18,19,20]. These microbial communities can be fueled by chemolithotrophic processes [21,22,23]. In some marine areas with low respiration, O_2_ penetrates to a great depth in sediments and rocks [24,25], which allows for the presence of aerobic microorganisms, including Thaumarchaeota, in the marine deep subsurface. High abundances of Thaumarchaeota have also been found in deep terrestrial subsurface—including interior rock samples of the Deccan Traps, India, from depths of less than 100 m to as much as 1400 m [26]; and fissure waters from gold mines in South Africa at depths of up to 3.08 km [27]. In 2005, high abundances of archaea, which were mainly assigned to Crenarchaeota, were reported in metamorphic rock samples with depth of great than 2000 m from the Chinese Continental Scientific Drilling Project (CCSD) in eastern China [28], and most of the crenarchaeotal lineages were proved to be Thaumarchaeota in that study. Here, we also present the high abundance of Thaumarchaeota in biofilm growing on supporting pipe at a depth of 2000 m from the CCSD well. These results indicated that the environmental conditions, especially availabilities of O_2_ and chemical energy, were suitable for Thaumarcheaota involved bio-geochemical processes in the deep metamorphic subsurface in eastern China.

## 2. Materials and Methods

### 2.1. Site Description and Sample Collection

The CCSD well (34°24′20.9″ N, 118°40′22.1″ E) is located in eastern China near the Tan-Lu fault, drilled into the Dabie-Sulu metamorphic terrain at a depth of 5158 m [29]. Paragneiss, eclogite, orthogneiss, and gneiss comprise the majority of the terrain, which were developed with fractures [30]. The geothermal gradient at the site is approximately 25 °C/km [29]. After completion in 2005, the well was used as a long-term observation site by installing seismometers, geothermometers, and pressure gauges at 4 levels, all of which were supported by drilling pipes. In 2015, the downhole instruments were raised up for maintenance, and visible biofilm was found attached to the supporting pipe at a depth of around 2000 m. A sample of this biofilm was collected into a sterile bag and stored at −20 °C until processing.

Fluid composition of the deep subsurface was measured with the drilling fluid during the initial drilling and rock sampling [28,31]. CH_4_, CO_2_, and He were identified as naturally occurring in the deep geological fluid. The measured high values of O_2_ (higher than 2%) and N_2_ (higher than 10%) in drilling fluid could not be attributed to either air or deep subsurface sources [30]. Anions in pore water from the rock samples included F^−^, Cl^−^, NO_3_^−^, and SO_4_^2−^, among which, the concentration of NO_3_^−^ was the highest, reaching 15 mg/g rock, an order of magnitude higher than that of SO_4_^2−^ [28]. Such a high concentration of NO_3_^−^ precluded the possibility of contamination by drilling fluid.

### 2.2. Morphological Observation of Microbes in Biofilm

Scanning electron microscopy (SEM) was employed to observe any morphological characteristics of microbes in the biofilm sample. Briefly, the biofilm was spread onto an ethanol-cleaned glass cover slip that was pre-treated with poly-L-lysine as an adhesive. Cover slips with deposited samples were fixed with 2% paraformaldehyde and 2.5% glutaraldehyde followed by sequential dehydration with ethanol, followed by critical point drying with a Quorum K850 Critical Point Dryer, and Pt coating with a Quorum SC7620 Sputter Coater [32]. Morphological observations were made using a field emission SEM (SUPRA 55, Zeiss, Oberkochen, Germany).

### 2.3. DNA Extraction from Biofilm, Amplification of 16S rRNA Genes, and Sequencing

The biofilm sample was divided into subsamples. Total DNA was extracted using FastDNA SPIN Kit for Soil (MP Biomedical, Solon, OH, USA) with a final elution in 70 µL de-ionized water following the protocol of our previous study [13]. To amplify the V4 hypervariable region of the prokaryotic 16S rRNA gene of bacteria and archaea, the primer set 515F (5′-CAGCMGCCGCGGTAA-3′) and 806R (5′-GGACTACHVGGGTWTCTAAT-3′) was used and 8 bp barcodes were added to the 5′-end of the primers to distinguish different samples. The polymerase chain reaction (PCR) system and amplification procedure followed those of Hou et al. [13]. Sequencing was conducted on an Illumina Hiseq platform at Magigene Biotechnology Co., Ltd. (Guangzhou, China).

### 2.4. Microbial Community Analyses

The raw data obtained by sequencing were analyzed on a public Galaxy Pipeline platform (http://mem.rcees.ac.cn:8080, 25 January 2022) [33]. Firstly, the barcode was used to assign sequences to the different samples, after which the barcode and the primers from both ends were removed [34]. FLASH was used to combine the reads [35]. The maximum overlap length was 250 and the minimum was 30. After filtering out low quality sequences (those with mean quality score lower than 30), DADA2 was used to generate an amplicon sequence variant (ASV) table with a similarity level of 97% [36]. Finally, the ASVs were taxonomically assigned by using RDP Classifier [37]. The microbial functions regarding C, N, and S cycles were predicted by FAProTax (Version 1.2.4) based on the ASV table [38].

### 2.5. Phylogenetic Analysis of Phylum Thaumarchaeota from the Deep Subsurface

To construct a phylogenetic tree of Thaumarchaeota sequences from this study, other thaumarchaeotal 16S rRNA gene sequences from the deep subsurface were also retrieved from the NCBI database. These sequence sources included fissure water and mine service water from gold mines in South Africa, with depths ranging from 0.7 to 3.08 km [27]; igneous rock from a borehole drilling project in the Deccan Traps in India, with depths ranging from ~60 to ~1400 m [26]; and metamorphic rocks from the CCSD borehole drilling project in eastern China [28,39]. The sequences from the Deccan Traps subsurface rock samples were produced by Illumina MiSeq sequencing, and the representative Thaumarchaeota sequences were obtained with DADA2 algorithm [36]. The sequences from the South African gold mines water samples and rock samples from the CCSD drilling project in China were produced by clone sequencing, and were assigned to uncultured Crenarchaeota, other than Thaumarchaeota [27,28,39]. All thaumarchaeotal 16S rRNA gene sequences were cut to the same length with Cutadapt after alignment [34]. UPGMA clustering based on the Jukes–Cantor distance model (including 1000 bootstrap replicates) was used to construct Thaumarchaeota lineages from the deep subsurface.

## 3. Results and Discussion

### 3.1. Microbial Community in the Biofilm Sample

**Figure 1:**
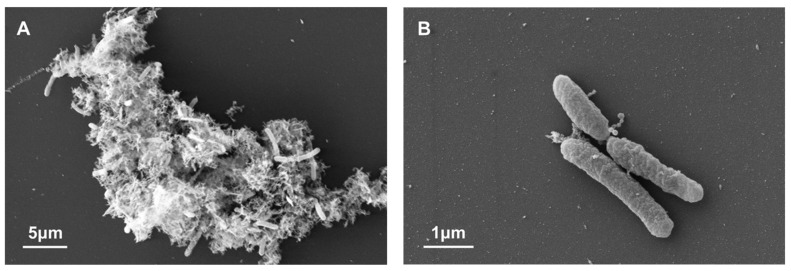
SEM images microbes in biofilm from the CCSD well. (**A**) Microbes which associated with each other via non-microbial substances; (**B**) Free-living microbes.

SEM observation showed that the majority of microbes were associated with each other via non-microbial substances (Figure 1A), with only a small fraction of free-living microbes observed (Figure 1B). Rod-shaped cells dominated the community. Formation of the biofilm suggested a high abundance of biomass in the metamorphic rock of the deep subsurface in eastern China, which was consistent with previous studies of samples from the same borehole [28,39,40]. In CCSD rocks, the biomass was generally higher than 10^4^ cell/g rock after staining of high porosity rocks with 4′,6-diamidino-2-phenylindole (DAPI) [40]. In some rock samples, the biomass exceeded more than 10^8^ cell/g rock as inferred by PLFA concentrations [28,39]. In contrast, the cell concentrations in deep subsurface rocks from the drilling projects in the Deccan Traps, India, were only 10^3^–10^4^ archaeal cells per gram of rock [26].

**Figure 2:**
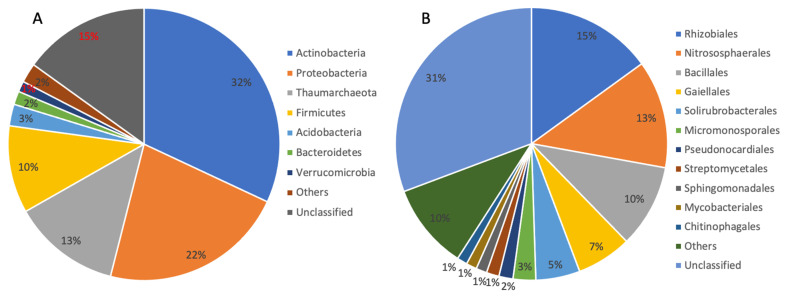
Relative microbial abundances at the level of phylum (**A**) and order (**B**). Only the taxonomic groups with relative abundances greater than 1% are displayed.

High-throughput sequencing was used to reveal the microbial community of the biofilm sample obtained from the CCSD borehole. A total of 59,255 raw sequences were obtained from two sequencing replicates. A total of 41,991 clean sequences were grouped into 668 ASVs, and assigned to 20 phyla, 60 classes, 97 orders, 158 families, and 234 genera. These bacterial and archaeal lineages matched microbial groups observed in previous clone-sequences of rock samples from the CCSD well [28,39,40]. Within the biofilm, 12.9% of sequences could be assigned to Archaea, 85.6% to Bacteria, and the remaining were unclassified. At the phylum level, Actinobacteria, Proteobacteria, Firmicutes, Acidobacteria, Bacteroidetes, and Verrucomicrobia were the major bacterial groups (relative abundance greater than 1%, Figure 2A). Thaumarchaeota was the major archaeal phylum (Figure 2A). As previously noted, the phylum Thaumarchaeota was assigned to Crenarchaeota in earlier publications with rock samples from the CCSD borehole [28,39]. Rhizobiales within the phylum Proteobacteria, Nitrosophaerales within the phylum Thaumarchaeota, and Bacillales within the phylum Firmicutes were the top three orders (Figure 2B). Many members of the above phyla are known to grow aerobically. These aerobic members included Thaumarchaeota (proved to be AOA in the following phylogenetic analysis, about 12.8% in relative abundance in the biofilm sample) [41,42], *Gaiella* (6.6%) [43], *Methylobacterium* (6%) [44], and *Solirubrobacter* (1.7%) [45]. The high abundance of aerobes distinguish the microbial community in the CCSD biofilm sample from most anaerobe-dominated microbial communities of the continental subsurface [46,47,48].

### 3.2. Thaumarchaeota Diversity in the Deep Subsurface

**Figure 3:**
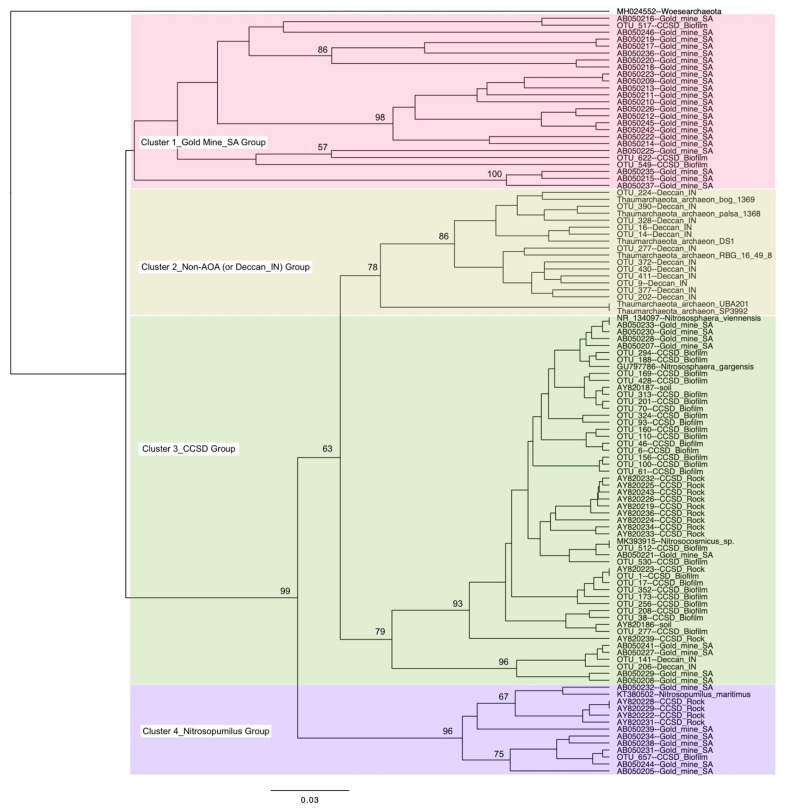
UPGMA tree of Thaumarchaeota based on the V4 variable region of 16S rRNA gene. Sequences from South African gold mines [27], Deccan Trap rock samples from India [26], and rock samples from CCSD drilling [28] were also included, which were denoted with Gold_mine_SA, Deccan_IN, and CCSD_Rock, respectively. The thaumarchaeotal sequences obtained in this study were denoted with CCSD_Biofilm. Bootstrap values higher than 50% were labeled on the nodes. Different colors denote the four clusters.

There have been few studies showing a high relative abundance of Thaumarchaeota in the deep subsurface, because of the generally anoxic condition of this habitat. To our knowledge, prior to our study, a high relative abundance of Thaumarchaeota has only been reported in rock samples from the Deccan Traps in India [26] and water samples from gold mines in South Africa [27]. Some members of Thaumarchaeota are not AOA, consisting of anaerobes or facultative aerobes [49,50]. A phylogenetic tree based on the 16S rRNA gene was constructed to inspect the relationship of these thaumarchaeotal sequences from the deep subsurface with non-AOA sequences (Figure 3). The Thaumarchaeota sequences could be divided into four clusters on the phylogenetic tree. Cluster 1 mainly included the sequences from the South African gold mines, with relatively long phylogenetic distances. Cluster 2 contained the majority of sequences from the Deccan Traps [26] and all non-AOA sequences were obtained from previous publications [3,51], which suggested that the Deccan Traps Thaumarchaeota may be anaerobes or facultative aerobes. Cluster 3 mainly consisted of the sequences from the rocks and biofilm from the CCSD drilling. These sequences were closely related to the AOA genera of *Nitrososphaera* and *Nitrosocosmicus*, which suggested these CCSD sequences were true AOAs. Cluster 4 contained some sequences from CCSD and South African gold mines, which were related to the AOA genus *Nitrosopumilus*. The close relatedness of CCSD sequences to AOA sequences suggested that the deep subsurface in and around the CCSD well was oxic.

The CCSD well is located near to the Tan-Lu fault, which is a major fault in eastern China [29]. The fault potentially acts as a channel for mass-exchange between the surface and subsurface, with seismic activities along the fault promoting such exchanges, i.e., downward oxygen diffusion. The extremely low organic carbon content detected in CCSD rock samples indicated that low oxygen consumption may also be an important cause for the downward diffusion of oxygen to the deep subsurface [28].

### 3.3. Potential Nitrogen Cycling in the Deep Subsurface

**Figure 4:**
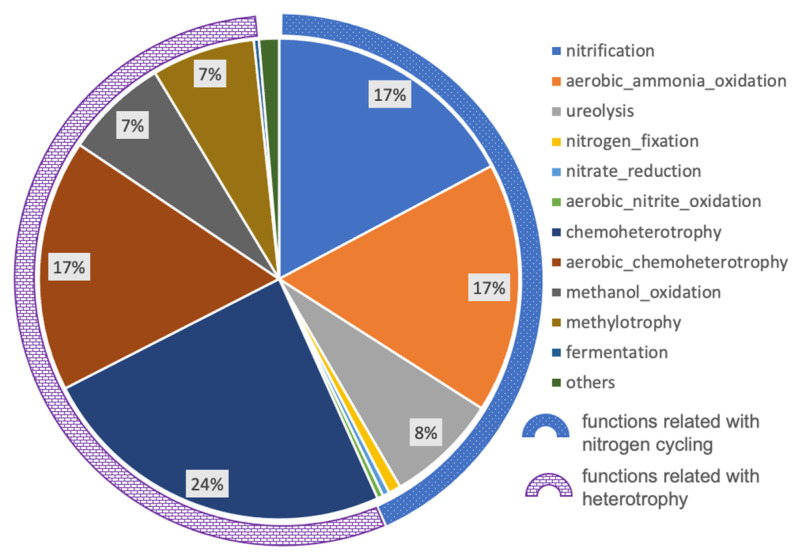
The relative abundance of major functional groups distribution (higher than 3‰). The blue dotted-arc indicates the functional groups related to nitrogen cycling, and the purple bricked-arc indicates the functional groups related to heterotrophy.

A total of 37 functional groups were obtained using FAProTax based on 144 out of 668 ASVs. The remaining 524 ASVs could not be assigned to any functional groups. The functionally predicted ASVs accounted for 33.3% of the total sequencing reads, which meant that a large fraction of microbial representatives were functionally unknown. Nevertheless, these predicted functional groups could still provide some clues about biogeochemical processes related with the growth of Thaumarchaeota, especially for nitrogen cycling. The relative abundances of predicted microbial community functional groups were calculated as the ASVs’ cumulative abundance allocated to each functional group (Figure 4). The major functional groups (relative abundances higher than 3‰) included nitrification, aerobic ammonia oxidation, ureolysis, nitrogen fixation, nitrate reduction, aerobic nitrite oxidation, chemoheterotrophy, aerobic chemoheterotrophy, methanol oxidation, methylotrophy, and fermentation. The former six functional groups were related to nitrogen cycling, accounting for 43.3% of total abundance, while the latter five functional groups were related to heterotrophy, accounting for 55.0%. The remaining predicted functional groups included nitrogen respiration, nitrate respiration, anammox, iron respiration, sulfate respiration, methanogenesis, etc., most of which could also be assigned to nitrogen cycling and heterotrophy.

High relative abundances of predicted nitrogen-related functional groups, especially nitrification, suggested that nitrogen cycling potentially contributes a substantial portion of energy to the microbial ecosystem in the deep metamorphic rock around the CCSD well. Aerobic ammonia oxidation is only performed by species from the archaeal phylum Thaumarchaeota, which oxidize ammonia to nitrite, and the nitrite could then be aerobically oxidized to nitrate, which could be performed by members of the bacterial phylum Nitrospirae. These processes may be the cause of the high concentration of nitrate detected in rocks from the CCSD drilling, up to 8 mg/g rock in average, which was 1–3 orders higher than chloride and sulfate anions [28]. The source of ammonia becomes the key question for the nitrification process. Within the biofilm, a high abundance of potentially diazotrophic bacteria, i.e., members from the order Rhizobiales (relative abundance of 15%, Figure 2), were detected. These bacteria included *Bradyrhizobium*, *Mesorhizobium*, and *Methylobacterium* which may aerobically fix nitrogen in a free-living life style [52,53] and contribute ammonia to the system, although some free-living non-diazotrophic rhizobia were reported recently [54]. Rhizobiales are also heterotrophs, so the autotrophic members, such as Thaumarchaeota and Nitrospirae, may provide organic matters in return. Some diazotrophic bacteria, such as *Methylobacterium*, are methylotrophic [55], and may utilize CH_4_ as their energy source, as CH_4_ was detected in the CCSD well [31].

## 4. Conclusions

AOA within Thaumarchaeota are an important group of microorganisms on Earth, especially in relation to carbon and nitrogen cycling. High-throughput sequencing of 16S rRNA gene revealed a high relative abundance of Thaumarchaeota in the deep terrestrial subsurface of eastern China. Phylogenetic analyses showed that these Thaumarchaeota representatives were AOAs and that oxic conditions were present in the deep subsurface (about 2000 m). The local geological settings may be the reason for such oxic conditions—i.e., the surface–subsurface connectivity along the Tan-Lu fault and its seismic activities may have brought oxygen to such depths. The reaction between downward diffusing O_2_ and upward diffusing CH_4_ potentially fuels the ecosystem with a high relative abundance of Thaumarchaeota.

## Data Availability

The raw sequence data reported in this paper have been deposited in the Genome Sequence Archive in National Genomics Data Center, Beijing Institute of Genomics (China National Center for Bioinformation), Chinese Academy of Sciences, under accession number CRA005978. They are publicly accessible at https://bigd.big.ac.cn/gsa, 28 January 2022.
